# Impact of maternal nationality on caesarean section rate variation in a high-income country

**DOI:** 10.5339/qmj.2021.69

**Published:** 2021-11-28

**Authors:** Saheed Shittu, Lolwa Alansari, Fahed Nattouf, Tawa Olukade, Naji Abdallah

**Affiliations:** ^1^Department of Obstetrics and Gynaecology, Al-Wakra Hospital, Hamad Medical Corporation, Qatar E-mail: SShittu@hamad.qa; ^2^Department of Paediatrics, Hamad General Hospital, Hamad Medical Corporation, Qatar

**Keywords:** Maternal nationality, Caesarean section rate variation, Migrants, High-income country

## Abstract

Background: Caesarean section (CS) rates have been reported to differ between immigrants and native-born women in high-income countries.

Objective: We assessed the CS rate and its relationship with the CS rate in country of nationality and other explanatory factors among women of different nationalities including Qatari women who underwent deliveries at our hospital to generate evidence that will quantify and help explain the observed CS rates in our hospital.

Methods: In this retrospective cross-sectional study conducted at the second-largest public maternity hospital in Qatar, Al-Wakra Hospital (AWH), data for all births delivered in 2019 were retrieved from the hospital's electronic medical records. The CS rates and the crude and adjusted risks of Caesarean delivery for mothers from each nationality were determined, and the common indications for CS were analyzed based on nationality. The association between nationality and Caesarean delivery was examined using binomial logistic regression analysis, with Qatari women as the reference group. The correlation between CS rate in country of nationality and observed CS rates in Qatar was also examined using Pearson's correlation.

Results: The study population consisted of 4816 births by women of 68 nationalities, of which 4513 births were by women from 25 countries. The highest proportion of deliveries (n-1247, 25.9%) was by Indian women. The frequency of CS was the highest and lowest among Egyptian (49.6%) and Yemeni women (17.9%), respectively. Elective CS was predominantly performed in women of Arab nationalities; the most common indication was a history of previous multiple CSs. Emergency CS was primarily performed in women of Asian and Sub-Saharan African nationalities; the most common indications were failure to progress and fetal distress. For most nationalities, the CS rate in Qatar was associated with those of the countries of nationality.

Conclusions: The observed CS rates varied widely among women of different nationalities. The variation was influenced by maternal factors and medical indications as well as the CS rates in the country of nationality. We posit that cultural preferences, acculturation, and patient expectations influenced observed findings. More efforts are required to reduce primary CS rates and to help women make the most informed decisions regarding modes of delivery.

Key Message: CS rates varied widely among women of different nationalities. The variation was influenced by medical indications, maternal preferences, and CS rate in countries of nationality. The solution to reducing CS rates should be a culturally informed response.

## 1. Introduction

Caesarean section (CS) is a surgical procedure that has positively influenced feto-maternal outcomes worldwide, with CS accessibility and outcomes serving as proxy measures of the availability of emergency obstetric services and healthcare quality in many parts of the world.^1-2^ Although CS is considered a safe and beneficial procedure, it is not without risks and is associated with short- and long-term complications for both the mother and child. Moreover, the procedure is associated with increased healthcare costs in most countries, with the costs borne by either the individuals or the government.^3-6^ As a result, there have been professional and public health concerns regarding the rising rate of CS worldwide,^7^ including concerns regarding under- and over-use of the procedure, which are influenced by medical and non-medical reasons. The rise in CS rates has been attributed to numerous causes, including an increase in indications for the procedure, introduction of the cardiotocograph, improvement in surgical and anesthetic techniques, defensive medicine or fear of malpractice litigation, professional practice styles, respect for women's autonomy, socioeconomic and cultural factors, as well as economic and organizational factors.^7,8^

In 1985, to balance the risks and benefits of CS, the World Health Organization (WHO) and the United Nations Population Fund recommended a target CS rate of 5%–15% of deliveries based on the estimated number of births that required medically necessary CS.^9^ National CS rates above 15% were found to offer no additional benefits on maternal and neonatal outcomes, while rates below 5% were inadequate for reducing maternal and neonatal mortality and morbidity.^10-12^ Thus, national CS rates above 50% in Latin American countries such as Brazil and below 5% in Sub-Saharan Africa and South-East Asia are both concerning.^7-10^ In contrast, Nordic countries such as Norway have managed to achieve a combination of rather low CS rates of 15% to 17% of all live births and low perinatal and maternal mortality rates.^13^ In Qatar, the CS rate has gradually increased from 16.3% in 1998 to 29.8% in 2013.^14^

The increasing economic disparities between poor and wealthy nations have resulted in a considerable increase in international migration, with women representing approximately 50% of all migrants globally.^15,16^ In many Western European countries, approximately one-fifth of all births are by immigrant women.^15^ The remarkable economic development in Gulf countries over the last few decades has drawn both skilled and semiskilled workers to various sectors of the economies, with a considerable proportion of these workers being women of reproductive age.^15,16^ Several studies have shown disparities in the number of Caesarean births to migrant women and nationals of industrialized countries.^8,15-18^ CS rates were higher in some nationalities and lower in others, and several factors were shown to influence the observed CS rates in the immigrant population.^15,20-24,30^ Thus, considering the worldwide increase in CS rates and the reported variation of same among immigrants in other studies, we assessed the CS rate and its relationship with those in the countries of nationality and other explanatory factors among women of different nationalities who underwent deliveries in our hospital setting to quantify and explain our observed CS rate.

## 2. Methods

### 2.1. Study design

We conducted a one-year retrospective cross-sectional study of deliveries conducted at Al-Wakra Hospital (AWH), Qatar, in 2019 with the help of the medical records department. Maternal electronic records were reviewed, and the study data were collected for analysis using Microsoft Excel Spreadsheet. The study was approved by the Hamad Medical Corporation Institutional Review Board (HMC-IRB Reference: MRC -01-21/054).

#### 2.2.1. Setting and participants

Qatar, which has one of the highest gross domestic product per capita values in the world, is home to a large economic migrant population, predominantly composed of Arabs, Asians, and other nationalities. Qatar has a population of 2.9 million, of whom 25% are females, with the vast majority living in Doha, the capital. Foreign workers amount to approximately 90% of the population, with the Indians being the largest community. Qataris constitute only 10% of the country's total population, followed by other Arabs (13%), Indians (21.8%), Filipino (7.35%), Nepali (12.5%), Bangladeshis (12.5%), Egyptians (9.35%), and Sri Lankans (4.35%).^17^ The population for this study consisted of women who underwent deliveries between January and December 2019 at the second-largest public maternity hospital in the country. The hospital receives approximately 6000 deliveries annually. Healthcare is accessible and free or heavily subsidized for all citizens and residents, with almost 90% of all deliveries conducted at public hospitals.^14^ For this study, we retrieved the data for 4816 births at 24 or more weeks of gestation and excluded births below 24 weeks

#### 2.2.2. Patient and public involvement

This study is a retrospective analysis of data. Patients or members of the public were not directly involved in design, conduct, reporting, or dissemination of our work.

### 2.3. Outcome

The main outcome was delivery by CS, with vaginal delivery (including normal and operative vaginal deliveries) serving as reference.

### 2.4. Exposure

The main exposure was maternal country of nationality, which was based on the nationality linked to each woman's international passport or national identification document. Women from other nationalities were examined in comparison to the Qatari women. In some cases, nationalities in close geographical proximity or sharing similar birth characteristics were merged into one national group, while all other nationalities with fewer than 30 births were grouped as “other nationalities’’. The grouped nationalities included Bangladeshi and Sri Lankan, Ethiopian and Eritrean, and Jordanian and Palestinian.

### 2.5. Other covariates

Maternal age at delivery was categorized as ≤ 24 years, 25–34years, or ≥ 35 years. Parity was categorized as nulliparous, parous with no previous CS, or parous with previous CS. Maternal height was coded as < 150 cm or ≥ 150 cm. However, height was included as a continuous variable in the analysis due to empty cell categories for some nationalities. We also included body mass index at delivery as a continuous variable. Other dichotomous covariates included the type of pregnancy (singleton/twin) and timing of birth (preterm or term delivery, with gestational age at delivery 24–36 weeks or ≥ 37 weeks, respectively). Maternal medical conditions, including diabetes and hypertensive diseases, were also evaluated. The indications for CS were classified based on their prevalence and if the CS was elective or emergency. For elective CS, we included cases with a previous CS needing another CS for medical indication, a previous CS refusing VBAC (vaginal birth after previous Caesarean), previous multiple CSs, breech presentation, multiple pregnancies, and others. For emergency CS, indications were classified as fetal distress, failure to progress, failed induction of labor, a previous CS refusing VBAC in labor and others. CS rates in country of nationality were retrieved from the UNICEF data warehouse.^32^ Delivery outcome was categorized as live birth or stillbirth, while birth weight was categorized as low birth weight ( < 2500 g) or normal birth weight ( ≥ 2500 g).

### 2.6. Statistical analysis

We summarized the distribution of data using numbers and percentages, and means and standard deviations, as appropriate, and examined the correlation between CS rates in the countries of nationality and the observed CS rates using Pearson's correlation coefficient values. We also examined the association between nationality and Caesarean delivery by using binomial logistic regression analysis with Qatari women as the reference group. The regression model was separately adjusted for maternal age, parity, preterm delivery, low birth weight, medical complications (including hypertensive disorders and diabetes), maternal height, and delivery BMI to examine the extent to which these factors could explain ethnic differences in the risk of CS based on previous research.^15^ Estimated crude and adjusted odds ratios (ORs) were reported with p-values. Statistical analysis was performed using IBM SPSS 26 statistical software, with statistical significance set at p < 0.05.

## 3. Results

Overall, we examined 4816 births from 322 (6.7%) Qatari women and 4494 (93.3%) women of 68 other different nationalities. Table 1 presents the characteristics of the study population. The highest proportion of deliveries (n = 1247, 25.9%) was from Indian women. The mean age of the women was 29.7 ± 5.1 years, and approximately two-thirds (66.5%) were aged between 25 and 34 years. The average height was 159.4 ± 6.2 cm, with less than 5% of the women shorter than 150 cm. More than a quarter of these women (27%) had undergone a CS prior to the current pregnancy. The overall CS rate was 36.7%, (elective CS, 22.4%; emergency CS, 14.3%). Primary CS rate was 16.9%. There were 442 operative vaginal deliveries (Vacuum = 363, 7.54% and forceps = 79, 1.64%). Most of the births were from singleton pregnancies, resulting in largely term (93%) live births (99.9%) and normal birth-weight babies (92.6%).

### 3.1. Characteristics of births by maternal nationality and other covariates

Maternal characteristics and births varied among nationalities. The mean maternal age ranged from 28 ± 6 years (Yemeni) to 32 ± 4 years (Filipino) (Table 2). The Qatari population included the highest proportion of women greater than 34 years of age (29.5%), followed by the Filipino (27.7%), Eritrean and Ethiopian (25.6%), Egyptian and GCC (27.3%), and Sudanese (23.4%) populations. In contrast, the Indian (10.7%) and Nepalese (12.9%) populations had the lowest proportions of older women. The mean heights ranged from 155 ± 4.6 cm (Nepalese) to 165 ± 5.9 cm (Nigerian). Approximately 10% of the Filipino and Nepalese women were shorter than 150 cm.

More than half of the Tunisian and Moroccan women and approximately two-fifths of Eritrean and Ethiopian, Filipino, Nigerian, and Nepalese women were nulliparous. A history of CS greater than 30% was observed in the following nationalities: Egyptian (43.8%), Lebanese (33.3%), Syrian (30.4%), Bangladesh/Sri Lankan (30.5%), and Indian (31%). A further breakdown of the history of previous CS showed that approximately a quarter of Egyptian women (27%) had previous multiple CSs; the corresponding proportions in the Nepalese, Lebanese, Qatari, Syrian, and Sudanese populations were above 10%, while only about 2% of Eritrean and Ethiopian women had previous multiple CSs (Tables 2a and 2b ). The incidence of gestational hypertensive disorders was the highest among Nigerian (12.2%), Filipino (8.7%), Bangladesh/Sri Lankan and Sudanese (8%), and Ethiopian/Eritrean (7%) women, and the least in Moroccan (2.9%), Qatari (2.8%), Yemeni (2.7%), and Syrian women (1.6%). In addition, approximately half of the Bangladeshi/Sri Lankan, and Nepalese women had diabetes during pregnancy, while the lowest prevalences of diabetes were observed in the Moroccan (25.7%), Algerian (23.5%), and Syrian (22.6%) women (Table 2b)

### 3.2. Indications for CS overall and based on nationalities

**Figure 1.**
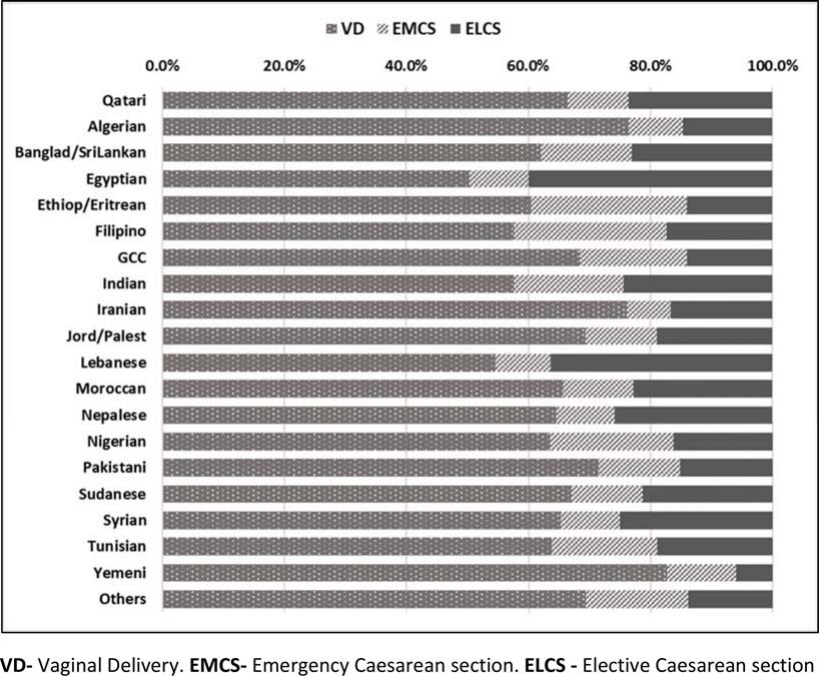
Distribution of delivery mode for each nationality

**Figure 2.**
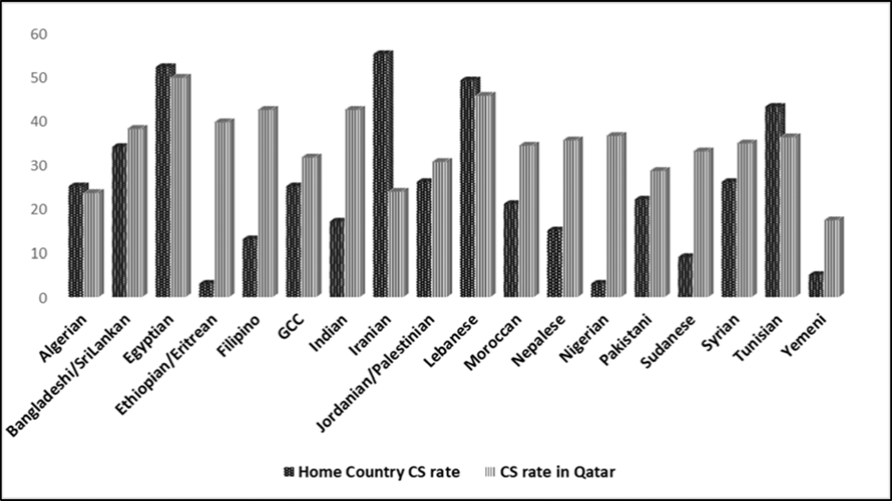
Maternal CS rate (%) in countries of nationality versus those in Al-Wakra Hospital.

A CS was performed in 1767 (36.7%) women; 1078 (22.4%) of the births were planned elective CS and 688 (14.3%) were performed as emergency CS. Primary CS rate was 16.9% (594/3517). The nationalities with the highest CS rates were Egyptian (49.6%), Lebanese (45.5%), and Filipino and Indian (both 42.2%) as shown in Figures 1 and 2. The CS rate for the Qataris was 33.4%. The most common indication for elective CS was previous multiple CS (39.9%) and one previous CS refusing VBAC (26.8%). The Egyptian population showed the highest proportion of women with previous multiple CSs (23%) as shown in Table 3. Refusal to try VBAC was the highest among Indians and least among the Qataris. The most common indications for emergency CS were fetal distress (30.2%) and failure to progress (28.4%), and were more common among women from the Philippines, Nigeria, and Ethiopia/Eritrea. 10.5% of the women who had emergency CS, qualified for VBAC but still opted for CS even though they were in labor (Table S1).

### 3.3. Maternal nationality, country of nationality, and host-country CS rates

Overall, the estimated host-country CS rates were high for all nationalities (17.3% to 49.7%) (2). The rates for 12 nationalities ranged from 30.6% to 39.5%, while those for Pakistani, Iranian, and Algerian ranged between 23.5% and 28.5%. Women from countries with high CS rates maintained high rates in Qatar, while those from countries with low CS rates also showed high rates in Qatar. The CS rate in country of nationality versus host-country rates were markedly different in Nigerians (3% vs. 36.5%), Yemenis (5% vs. 17%), Ethiopians/Eritreans (3% vs. 39.6%), and Sudanese (9% vs. 33%). A statistically significant weak linear relationship was observed between the CS rate in country of nationality and observed host-country rates (r = .354, p <  .001).

### 3.4. The association between maternal nationality and other covariates

When compared to Qatari women, Egyptian, Filipino, and Indian women were significantly more likely to undergo CS (crude ORs, 1.95, 1.45, and 1.45, respectively; p <  0.05) (Table 4). When adjusted for other covariates, Egyptian women were twice as likely to undergo CS (adjusted for maternal age, preterm delivery, height, delivery BMI, hypertensive status, and diabetic disorders). Likewise, Indian women were approximately 1.5 times more likely to undergo CS for similar factors. The risk for Filipino women was similar to that of Indian women but attenuated for maternal age, height, and hypertensive disorders. Crude and adjusted ORs for Yemeni women showed a significantly consistent 44% to 61% decrease in the likelihood of CS delivery. No significant crude or adjusted ORs were observed for other nationalities.

## 4. Discussion

CS rates were generally high among all nationalities in our setting, and the indications for CS were closely linked to maternal nationality. In addition, women from nationalities with high CS rates had equally high CS rates in Qatar. This hospital setting catered to a high number of immigrant women of reproductive age, with approximately 7–8 of every 10 women aged less than 35 years old or showing a previous parous experience. About a quarter of these women also had a previous CS.

Elective CS was largely performed in women of Arab nationalities, while emergency CS was more prominent in Asian and Sub-Saharan African nationalities. The most common indication for elective CS was multiple prior CS; this was most prominent among Egyptians. Conversely, failure to progress and fetal distress were the most common indications for emergency CS and were most prominent among women from the Philippines. Notably, the Filipino population included approximately 10% of women with height of less than 150 cm. It had the highest mean age and the second highest incidence of hypertensive disorders, resulting in a quarter of the women of this nationality receiving emergency CS. The effect of stature as an explanatory factor for increased CS rate among these women has been noted in other studies.^8,15,27-28^

The literature suggests that the CS rates among immigrants vary with that of the host countries and other migration indicators, including country of birth, length of time in the host country, and migration classification (for economic migrants and refugees).^21-26-30^ The literature lists several risks faced by migrant pregnant women that worsen their childbearing health risks, including discrimination, social isolation, lack of support, high-level stress related to migration and resettlement, poverty, limited or no health insurance, violence and trauma, and barriers to accessing health care.^19,20,21^ However, evidence to explain high CS rates among migrants is lacking. The pathway for higher CS rates among migrants may be related to poor maternal health due to infections, malnutrition, and anemia; higher prevalence of high BMI and gestational diabetes; women's cultural attitudes and expectations regarding labor and delivery management; genital cutting; language and cultural barriers; quality of care; and acculturation,^30^ whereby migrant women associate CS with better care as they adopt the attitudes, expectations, behaviors, and traditions of the receiving countries.

Since Qatar offers universal and free or heavily subsidized access to high-quality care and delivery, we theorize that the CS rates in the countries of nationality or acculturation had a significant influence on the observed CS rates in this study. We observed that women from countries with high CS rates often had histories of previous CS and either declined VBAC or were eligible for elective CS. In contrast, women from countries with low CS rates either required emergency CS or acculturated and had a lower threshold for CS intervention. The fact that 80% of the Iranian women were nulliparous or had no CS history could explain the marked difference from the CS rate in Iran, which is known to be quite high (55%).^32^ The relatively low percentage of elective CS and high percentage of emergency CS among Ethiopian and Eritrean women reflect the quest for vaginal birth and strong resistance to CS among these women, which is well documented in the literature.^25^ The high CS rate among Egyptians has been linked to various reasons in Egypt including tendency to deliver in private setting and maternal request due to fear of pain, prolonged labor and genital laceration.^26^ This may also explain why their proportion of women with history of CS is highest (43.8%) in our hospital (Table 2a).

Similar studies done in other high-income countries with multi-ethnic population as in Spain, Norway Portugal and Belgium also showed variation in CS rates between women from different nationalities.^8,18,19,21^ Based on our results, our primary CS rate of 16.9 % is comparable to the WHO target of 10%–15%. The major contributors to the overall CS rate of 36.7% are women with previous multiple CSs and one previous CS refusing VBAC; hence, medical reasons are still the most important indications for CS in our setting, however a substantial share is due to maternal preference among those with one previous CS as shown in tables S1.

### 4.1. Implications for policy and practice

Our result showed that CS rate was high in AWH, with considerable variations among nationalities and that previous CS is the commonest indication for CS. The most recent WHO statement recommends providing CS to women in need of it rather than striving to achieve specific rates.^10-13^

Our effort to reduce CS rates should therefore concentrate on women without previous CS or those with one previous CS who are also suitable for vaginal birth after a previous CS (VBAC). An appropriate rate can be achieved in those without previous CS by preventing unnecessary primary CSs through implementation of various non-clinical interventions to reduce CS rates, such as adherence to departmental guidelines on labor induction and management, obtaining second opinions for CS indications, rigorous review of primary CS, and provision of appropriate feedback.^33^ For women with one prior CS, it is imperative to develop a pro-VBAC culture to promote and improve uptake of VBAC, which remains a safe and effective mechanism for reducing CS rates.^35^

Various recommendations suggested in the literature to reduce CS among immigrants in high-income countries^30^ will also improve care of such patients in our setting. These include improving access to prenatal care and education, provision of continuous support in labor through bilingual companions, and the use of professional interpreters and translated materials to achieve effective communication.

We suggest the need for more research into the existence of shared beliefs, behaviors, and cultural practices related to Caesarean delivery, to specify both the potential pathways of influence and how they may differ across nationalities.

### 4.2. Strengths and limitations

To our knowledge, this is the first study to examine the influence of maternal nationality on CS rate in Qatar. By examining all births during the study period and using routine data, our electronic health record system served as a surrogate registry for births during this period. This increased the statistical power of our findings, minimized selection bias, and increased the generalizability of the findings from our setting.

Our study had some limitations. It was a retrospective study and all the study data were from one hospital in Qatar. Findings from our hospital may not be generalizable to the entire country based on our sample size. Also, the modest size of our referenced Qatari group might limit some of the comparisons in the regression model. We did not include maternal socioeconomic status and educational level, which were not available for all patients in the electronic records. These are crucial factors that significantly affect maternal reproductive health choices.^30^ The use of routine health data is prone to measurement and misclassification bias and missing data from incomplete and variable documentation.^34^ Also, the small number of deliveries from some nationalities made it difficult to make general statements about them. Furthermore, the primary drivers of CS among our patients being previous multiple CSs and previous CS refusing VBAC constitute confounders for the influence of nationality as a predictor of CS. Finally, intra-country disparities have been noted in CS studies. As such, CS rates in countries of nationality used in this study could not account for such disparities^32,34^.

## Conclusion

The observed CS rates varied widely among women of different nationalities and were influenced by medical indications as well as the CS rates in the countries of nationality. We posit that cultural preferences, acculturation, and patient expectations influenced our observed findings. Women of Arab nationalities had predominantly elective CSs, while Asian and Sub-Saharan African women had more emergency procedures. More efforts are required to reduce unnecessary primary CS rates, promote VBAC, and help women make the most informed decisions regarding the routes of delivery. Our solution to high CS rates require a culturally informed response that will ensure equality and foster excellence in maternity care.

### Acknowledgments

We would like to thank Mr Renz Lupisan Guterriez from the Clinical Informatics Unit of Al-Wakra Hospital for data collection, and the Hamad Medical Corporation for funding of online publication.

### Declarations of interest

Authors report no conflicts of interest.

### Ethical Approval and Consent to participate

The study was approved by the Institutional Review Board of Hamad Medical Corporation (MRC-01-21-054). This was a retrospective study and the requirement for obtaining patient informed consent was waived. All procedures in this study were performed in accordance with the ethical standards of the institution and the 1964 Helsinki Declaration and its later amendment or comparable ethical standards.

### Consent for publication

Not applicable.

### Availability of data and material

The data that support the findings of this study are available from Hamad Medical Corporation and restrictions apply to the availability of the data due to institutional regulations.

### Source of Funding

Hamad Medical Corporation

### Contribution of Authors

LA conceived the study and contributed to study design. SS, FN, and NA conducted literature searches, participated in study protocol and conduct, data analysis, and drafted the manuscript. TO analyzed data and contributed to the draft manuscript. All authors read and approved the final version.

## Figures and Tables

**Table 1:** Characteristics of the study population

Characteristic	n = 4816	%
Age		
⩽24 yr	752	15.6
25–34 yr	3203	66.5
≥35 yr	861	17.9
Mode of delivery		
VD	3044	63.2
EMCS	693	14.4
ELCS	1079	22.4
Livebirth/Stillbirth		
Liveborn	4811	99.9
Stillborn	5	0.1
Preterm or Term		
24–36 wk	335	7
≥37 wk	4479	93
Missing data	2	
Birth weight		
≤2499 g	357	7.4
≥2500 g	4445	92.6
Missing data	14	
Height		
<150 cm	200	4.3
≥150 cm	4430	95.7
Missing data	186	
Parity		
Nulliparous	1348	28
Parous with no previous CS	2169	45
Parous with previous CS	1299	27
Pregnancy		
Singleton	4751	98.7
Multiple	65	1.3
	Mean	SD
Age (yr)	29.7	5.1
Birth weight (g)	3216	509.8
Height (cm)	159.4	6.2
GA at delivery (wk)	38.6	1.6
BMI at delivery	30.8	5.4

VD, vaginal delivery; EMCS, emergency caesarean section; ELCS, elective Caesarean section; CS, Caesarean section; GA, gestational age; BMI, body mass index

**Table 2a:** Characteristics of births by explanatory factors and maternal nationality

	Qatari	Algerian	Bangladesh/SriLankan	Egyptian	Ethiopian/Eritrean	Filipino	GCC	Indian	Iranian	Jordanian/Palestinian
	n=322	n=34	n=187	n=518	n=43	n=254	n=114	n=1247	n=84	n=259
Age										
(Mean ±SD)	31 ±6	30 ±5	29 ±5	31 ±5	31 ±5	32 ±4	29 ±6	30 ±4	30 ±5	30 ±5
≤24 yr	17.70%	11.80%	23.00%	10.60%	7.00%	0.80%	27.20%	10.60%	20.20%	11.60%
25–34 yr	52.80%	70.60%	59.40%	65.60%	67.40%	71.50%	49.10%	78.70%	61.90%	67.80%
≥35 yr	29.50%	17.60%	17.60%	23.70%	25.60%	27.70%	23.70%	10.70%	17.90%	20.50%
Parity										
Nulliparous	15.80%	32.40%	29.40%	16.80%	46.50%	40.30%	22.80%	28.10%	27.40%	20.20%
Parous with no previous CS	57.50%	50.00%	40.10%	39.40%	39.50%	38.30%	58.80%	40.90%	52.40%	56.20%
Parous with previous CS	26.70%	17.60%	30.50%	43.80%	14.00%	21.30%	18.40%	31.00%	20.20%	23.60%
Height										
(Mean ±SD)	159.1 ±5.7	164 ±5.7	157.1 ±5.4	161.2 ±5.5	160.6 ±5.7	155.2±5.3	158 ±5.6	158.2 ±6.	158.8 ±6.7	161.8 ±6.2
<150 cm	4.70%		7.30%	1.40%	2.40%	13.20%	5.50%	6.00%	8.90%	1.60%
≥150 cm	95.30%	100.00%	92.70%	98.60%	97.60%	86.80%	94.50%	94.00%	91.10%	98.40%
Singleton/Multiple										
Singleton	98.80%	97.10%	100.00%	98.50%	100.00%	99.60%	98.20%	99.00%	98.80%	98.40%
Twins	1.20%	2.90%		1.50%		0.40%	1.80%	1.00%	1.20%	1.60%
No. of Previous CSs										
0	73.30%	82.40%	69.50%	56.20%	86.00%	78.70%	81.60%	69.00%	79.80%	76.40%
1	12.70%	8.80%	21.90%	16.80%	11.60%	15.00%	11.40%	24.00%	11.90%	15.10%
>1	14.00%	8.80%	8.60%	27.00%	2.30%	6.30%	7.00%	7.00%	8.30%	8.50%
GDD										
No	68.00%	76.50%	46.50%	62.90%	55.80%	61.30%	70.20%	55.40%	65.50%	64.30%
Yes	32.00%	23.50%	53.50%	37.10%	44.20%	38.70%	29.80%	44.60%	34.50%	35.70%
Preeclampsia or PIH										
No	97.20%	94.10%	92.00%	96.10%	93.00%	91.30%	96.50%	94.50%	94.00%	95.70%
Yes	2.80%	5.90%	8.00%	3.90%	7.00%	8.70%	3.50%	5.50%	6.00%	4.30%
GA at birth										
24–36 wk	9.30%	5.90%	7.00%	5.80%	2.30%	9.10%	3.50%	7.40%	7.10%	5.80%
≥37 wk	90.70%	94.10%	93.00%	94.20%	97.70%	90.90%	96.50%	92.60%	92.90%	94.20%
Birth weight										
≤2499 g	9.00%	2.90%	8.60%	6.00%	4.70%	7.90%	7.10%	9.80%	9.50%	3.90%
≥2500 g	91.00%	97.10%	91.40%	94.00%	95.30%	92.10%	92.90%	90.20%	90.50%	96.10%
BMI at delivery										
Mean	32.1	31.1	29.8	33.3	28.4	29.3	31.1	30.2	30.7	31.7
Standard Deviation	6.8	5.7	4.4	5.5	3.9	5	6.4	4.7	5.4	5.9
Mode of delivery										
VD	66.50%	76.50%	62.00%	50.40%	60.50%	57.70%	68.40%	57.70%	76.20%	69.40%
CS	33.50%	23.50%	38.00%	49.60%	39.50%	42.30%	31.60%	42.30%	23.80%	30.60%

SD, standard deviation; CS, Caesarean section; GDD, gestational diabetes; PIH, pregnancy-induced hypertension; GA, gestational age; BMI, body mass index; VD, vaginal delivery

**Table 2b:** Characteristics of births by explanatory factors and maternal nationality continued

	Lebanese	Moroccan	Nepalese	Nigerian	Pakistani	Sudanese	Syrian	Tunisian	Yemeni	Others

	n = 33	n = 70	n = 31	n = 74	n = 640	n = 189	n = 319	n = 116	n = 151	n = 137

Age										

(Mean ± SD)	30 ± 5	29 ± 6	30 ± 5	30 ± 4	29 ± 6	29 ± 6	28 ± 5	30 ± 5	28 ± 6	31 ± 5

≤ 24 yr	15.20%	24.30%	12.90%	4.10%	23.60%	21.30%	27.60%	8.60%	30.70%	10.20%

25–34 yr	63.60%	58.60%	74.20%	82.40%	60.90%	55.30%	57.70%	75.90%	52.70%	68.60%

≥ 35 yr	21.20%	17.10%	12.90%	13.50%	15.50%	23.40%	14.70%	15.50%	16.70%	21.20%

Parity										

Nulliparous	21.20%	55.70%	45.20%	41.90%	28.00%	33.00%	23.50%	56.90%	31.30%	37.20%

Parous with no previous CS	45.50%	24.30%	25.80%	41.90%	50.50%	45.70%	46.10%	29.30%	56.00%	46.00%

Parous with previous CS	33.30%	20.00%	29.00%	16.20%	21.40%	21.30%	30.40%	13.80%	12.70%	16.80%

Height										

(Mean ± SD)	162.7 ± 5.5	162.6 ± 5.9	155 ± 4.6	165 ± 5.9	158.6 ± 5.4	162.6 ± 6.6	160.7 ± 5.7	163.8 ± 5.9	156.6 ± 5.4	161.4 ± 6.8

< 150 cm		1.60%	10.00%		3.60%	1.10%	1.00%		5.40%	3.80%

≥ 150 cm	100.00%	98.40%	90.00%	100.00%	96.40%	98.90%	99.00%	100.00%	94.60%	96.20%

Singleton/Multiple										

Singleton	100.00%	95.70%	100.00%	100.00%	97.80%	98.40%	98.40%	97.40%	99.30%	98.50%

Twins		4.30%			2.20%	1.60%	1.60%	2.60%	0.70%	1.50%

No. of Previous CSs										

0	66.70%	80.00%	71.00%	83.80%	78.60%	78.70%	69.60%	86.20%	87.30%	83.20%

1	18.20%	14.30%	9.70%	10.80%	12.50%	10.10%	17.20%	7.80%	10.70%	13.10%

>1	15.20%	5.70%	19.40%	5.40%	8.90%	11.20%	13.20%	6.00%	2.00%	3.60%

GDD										

No	60.60%	74.30%	48.40%	66.20%	62.00%	61.70%	77.40%	57.80%	60.00%	63.50%

Yes	39.40%	25.70%	51.60%	33.80%	38.00%	38.30%	22.60%	42.20%	40.00%	36.50%

Preeclampsia or PIH										

No	97.00%	97.10%	93.50%	87.80%	94.80%	92.00%	98.40%	96.60%	97.30%	93.40%

Yes	3.00%	2.90%	6.50%	12.20%	5.20%	8.00%	1.60%	3.40%	2.70%	6.60%

GA at birth										

24–36 wk	9.10%	8.60%	3.20%	8.10%	7.70%	5.90%	5.30%	6.00%	8.00%	5.10%

≥ 37 wk	90.90%	91.40%	96.80%	91.90%	92.30%	94.10%	94.70%	94.00%	92.00%	94.90%

Birth weight										

≤ 2499 g	6.10%	5.70%	3.20%	4.10%	8.30%	6.40%	3.80%	4.30%	6.80%	5.80%

≥ 2500 g	93.90%	94.30%	96.80%	95.90%	91.70%	93.60%	96.20%	95.70%	93.20%	94.20%

BMI at delivery										

Mean,	31	30	29.1	31.2	30.4	31.3	30.9	30.2	28.6	30.4

Standard Deviation	5.4	4.8	3.3	5.6	5.4	6.2	5.3	4.2	4.9	5.5

Mode of delivery										

VD	54.50%	65.70%	64.50%	63.50%	71.50%	67.00%	65.20%	63.80%	82.70%	69.30%

CS	45.50%	34.30%	35.50%	36.50%	28.50%	33.00%	34.80%	36.20%	17.30%	30.70%


SD, standard deviation; CS, Caesarean section; GDD, gestational diabetes; PIH, pregnancy-induced hypertension; GA, gestational age; BMI, body mass index; VD, vaginal delivery

**Table 3:** Indications for CS in relation to nationality (based on each country's total number of deliveries)

Indication for Elective CS	Qatar n - 322	Egypt n - 518	Philippines n - 256	India n - 1247

Previous CS and refused VBAC	3 (0.9%)	40 (7.7%)	14 (5.5%)	110 (8.8%)

Previous CS with obstetric indication	17 (5.3%)	27 (5.2%)	13 (5.1%)	83 (6.6%)

Multiple previous CSs	44 (13.6%)	122 (23%)	13 (5.1%)	73 (5.8%)

Breech presentation	6 (1.9%)	4 (0.8%)	3 (1.2%)	16 (1.8%)

Multiple pregnancies	3 (0.9%)	2 (0.4%)	0 (0%)	7 (0.6%)

Other	2 (0.6%)	8 (1.5%)	0 (0%)	17 (1.4%)

Indications for emergency CS	Qatar	Egypt	Philippines	India

Fetal distress	12 (3.6%)	15 (2.9%)	21 (8.2%)	90 (7.2%)

Failure to progress	4 (1.2%)	13 (2.5%)	21 (8.2%)	48 (3.8%)

Previous CS and refused VBAC	2 (0.6%)	1 (0.2%)	3 (1.2%)	12 (1%)

Failed IOL	1(0.3%)	2 (0.4%)	4 (1.6%)	7 (0.6%)

Others	13 (4%)	23 (4.4%)	16 (6.3%)	62 (5%)


CS, Caesarean section; VBAC, vaginal birth after CS; IOL, induction of labor

**Table 4:** Crude and adjusted odds ratios for the association between nationality and caesarean section

Nationality	Crude OR		Adjusted for Parity		Adjusted for preterm delivery		Adjusted for maternal age		Adjusted for low birth weight		Adjusted for height		Adjusted for delivery BMI		Adjusted for PET/PIH		Adjusted for GDD	

	OR	P	OR	P	OR	P	OR	P	OR	P	OR	P	OR	P	OR	P	OR	P

Qatari	1		1		1		1		1		1		1		1		1	

Algerian	0.61	0.24	0.57	0.285	0.62	0.266	0.62	0.257	0.64	0.297	0.75	0.496	0.73	0.461	0.59	0.213	0.61	0.246

Bangladeshi/Sri Lankan	1.21	0.313	0.9	0.661	1.24	0.265	1.33	0.147	1.22	0.300	1.12	0.575	1.47	0.054	1.16	0.440	1.19	0.359

Egyptian	1.95	0.000	1.28	0.219	2.02	0.000	1.95	0.000	2.04	0.000	2.14	0.000	1.94	0.000	1.94	0.000	1.94	0.000

Ethiopian/Eritrean	1.30	0.437	1.61	0.229	1.37	0.342	1.25	0.508	1.35	0.368	1.42	0.298	1.82	0.079	1.25	0.503	1.28	0.455

Filipino	1.45	0.032	1.53	0.055	1.46	0.030	1.34	0.093	1.47	0.026	1.27	0.180	1.85	0.001	1.38	0.062	1.44	0.034

GCC	0.91	0.702	1.20	0.535	0.96	0.859	0.99	0.959	0.94	0.801	0.89	0.620	1.02	0.923	0.91	0.681	0.92	0.707

Indian	1.45	0.004	1.21	0.286	1.48	0.003	1.53	0.002	1.46	0.004	1.42	0.009	1.78	0.000	1.42	0.007	1.44	0.006

Iranian	0.62	0.089	0.54	0.086	0.63	0.100	0.66	0.139	0.61	0.084	0.62	0.095	0.72	0.267	0.60	0.071	0.62	0.088

Jordanian/Palestinian	0.87	0.455	0.87	0.572	0.90	0.556	0.88	0.478	0.93	0.674	0.91	0.600	0.89	0.541	0.86	0.410	0.87	0.445

Lebanese	1.65	0.174	1.52	0.393	1.66	0.17	1.71	0.151	1.71	0.15	1.75	0.138	1.76	0.141	1.65	0.175	1.64	0.179

Moroccan	1.03	0.905	0.80	0.504	1.04	0.887	1.14	0.651	1.07	0.818	1.17	0.585	1.22	0.500	1.03	0.906	1.04	0.891

Nepalese	1.09	0.827	0.65	0.383	1.15	0.729	1.15	0.731	1.15	0.722	1.00	0.997	1.48	0.327	1.06	0.891	1.07	0.858

Nigerian	1.14	0.63	1.32	0.395	1.15	0.602	1.14	0.635	1.19	0.513	1.37	0.252	1.24	0.44	1.05	0.860	1.14	0.634

Pakistani	0.79	0.107	0.73	0.112	0.80	0.131	0.87	0.332	0.79	0.106	0.79	0.118	0.92	0.59	0.77	0.078	0.79	0.100

Sudanese	0.98	0.897	0.96	0.857	1.00	0.987	1.02	0.913	1.00	0.996	1.08	0.704	1.04	0.862	0.93	0.713	0.97	0.877

Syrian	1.06	0.737	0.78	0.267	1.10	0.575	1.20	0.286	1.11	0.530	1.12	0.516	1.21	0.261	1.07	0.688	1.07	0.704

Tunisian	1.12	0.604	1.15	0.619	1.16	0.522	1.14	0.562	1.18	0.476	1.35	0.200	1.34	0.204	1.12	0.621	1.12	0.629

Yemeni	0.42	0.000	0.45	0.008	0.42	0.000	0.46	0.002	0.43	0.001	0.39	0.000	0.56	0.022	0.41	0.000	0.41	0.000


Qatari women served as the reference nationality

BMI, body mass index; PET, pre-eclampsia; PIH, pregnancy-induced hypertension; GDD, gestational diabetes; OR, Odds ratio; P, P-value

Bold text indicates that the values are statistically significant at P <  0.05

**Table S1:** Indications for Caesarean Sections (% based on the total number of each type of CS)

Indications for elective CS	Frequency	Indications for emergency CS	Frequency

Previous multiple CS	39.9%	Fetal distress	30.2%

Previous CS and refused VBAC	26.8%	Failure to progress	28.4%

Previous CS with obstetric indication	21.05%	Previous CS and refused VBAC	10.5%

Breech presentation	5.4%	Failed IOL	9.2%

Multiple pregnancy	2.05%	Breech Presentation	6.3%

Other	4.8%	Others	15.4%


CS, Caesarean section; VBAC, vaginal birth after CS; IOL, induction of labor
